# A qualitative evaluation of a partnership between a local authority and community organisations to improve mental health and wellbeing

**DOI:** 10.1186/s12982-025-00875-1

**Published:** 2025-08-14

**Authors:** Judi Kidger, Berni Graham, Hannah Robinson, Chantelle Fatania

**Affiliations:** 1 Population Health Sciences, Bristol Medical School, University of Bristol, Canynge Hall, 39, Whatley Road, Bristol, BS8 2PS UK; 2 London, UK; 3 Haringey Council, Public Health, Level 4, 48 Station Road, London, N22 7TY UK

## Abstract

Mental health problems lead to earlier mortality, poor physical health and socioeconomic disadvantage. Some people, including those from black and minority ethnic groups and low-income households, are at higher risk of poor mental health and are less likely to seek support from statutory services. Community Protect (CP) is a model in which a local government public health team acquired funding to support third sector organisations working with marginalised populations. These organisations provided mental health ambassadors, social activities and signposting to mental health support. This qualitative study comprised 28 semi-structured interviews with those involved in delivering CP, and six observations of activities. Topic guides covered barriers and facilitators to CP delivery and perceived benefits. Analysis used the Framework Method. Five themes were identified: trust and collaboration; time; expectations and flexibility; the ambassador role and perceptions of impact. CP relied on trusting relationships built between the different organisations involved, and between organisations and their individual users. The limited timeframe imposed by the funding created a barrier to collaboration when planning CP, meaning it was not always sufficiently tailored to the target populations’ needs. Allowing organisations the flexibility to be responsive to their population’s needs was beneficial. Benefits included improved mental health and social connection for the users, and improved ability of the local public health team to reach marginalised groups. Community organisations play a key role in prevention and support for mental health problems among minoritised populations. For partnerships with statutory organisations to succeed, funding and timescales need to be realistic, and interventions developed collaboratively.

**Supplementary Information:**

The online version contains supplementary material available at 10.1186/s12982-025-00875-1.

## Background

Poor mental health affects large numbers of people every year and has far reaching consequences, including lower life expectancy and quality of life, poorer physical health, and higher risk of worse socioeconomic outcomes such as unemployment and homelessness [1–4]. In the UK, the latest survey data from 2023/24 estimated that 22.6% of adults over the age of 16 have a common mental health condition [1], and mental health problems are estimated to cost the UK economy at least £117.9 billion a year [5]. The distribution of population mental health conditions is unequal. Those from poorer backgrounds, with disabilities and chronic illness, certain minoritised ethnic groups and those who have minoritised sexual orientation and/or gender identities are at far greater risk of poor mental health outcomes, with evidence indicating these inequalities worsened during the COVID-19 pandemic [1, 6–9]. In the UK, less than half (47.7%) of those with a common mental disorder access mental health treatment [1] and those who are at higher risk of poor mental health experience particular barriers to accessing help, including services not being culturally appropriate and therapies that are not appropriate for people with learning disabilities [10, 11]. Further, the increasing demand on mental health services coupled with reduced budgets that has arisen in recent years is a particular challenge in more deprived areas [12].

Partly to address the inability of statutory mental health services to meet all needs, and because of the importance of prevention and early intervention to improve health and reduce inequalities [13], local authorities in the UK are viewed as having a key role in improving mental health and wellbeing [14]. Further, there has been a growing interest in partnership working with voluntary and third sector organisations, who often deliver the most innovative approaches to prevention and community-based mental health interventions [15, 16]. Poor mental health has a wide range of social determinants, such as socioeconomic disadvantage, discrimination and loneliness [17–19], and community-based interventions are potentially well placed to address these wider factors [16]. There is a small amount of evidence that support based in community settings can be effective at improving mental health [20–22]. A review of community based mental health interventions found positive effects on key outcomes such as self-esteem, physical functioning and social/life support, as well as depression and anxiety [22]. A number of key elements explain the potential of such interventions to address mental health needs, including those delivering the intervention being trustworthy, approachable and knowledgeable, the service itself being accessible, culturally appropriate, holistic and flexible, social connection being part of the intervention, and reduced stigma and a sense of safety in accessing support in familiar spaces [15, 21, 23–25]. Furthermore, interventions incorporating volunteer or peer support have demonstrated positive outcomes in improving wellbeing, alleviating symptoms of mental health conditions and reducing loneliness [22, 26]. The Covid-19 Community Champions model exemplifies partnership working between local authorities, voluntary and community sector (VCS) organisations, and community members to address health disparities during the pandemic. Qualitative research on the model highlights the critical role of trust between communities and public health bodies, the value of flexibility in approaches, and the need for tailored communication to meet the needs of diverse populations [27]. Effective partnerships were also found to rely on trust, clear communication, and mutual respect [28] but the sustainability of such partnerships may be threatened by insecure funding for VCS organisations [27, 28].

However, gaps remain in the evidence regarding the optimal ways to deliver such community-based interventions to marginalised populations and the underlying mechanisms by which they promote positive mental health and wellbeing [29–31]. This paper seeks to address that gap, by reporting findings from a qualitative study of a community-based intervention, commissioned by a local authority, which uses a partnership model to support mental health and wellbeing, specifically targeting groups experiencing disadvantage. We sought to answer two questions: (i) what are the barriers and facilitators to a community-led mental health partnership model working well? (ii) what are the perceived benefits to the organisations and individuals involved?

## Methods

### Setting

The local authority in which the intervention was delivered has a population of 264,200 people and was ranked as the fourth most deprived local authority in London in 2019 as measured by the Index of Multiple Deprivation score [32]. It has a highly diverse population, including sizeable populations with African, African Caribbean, East European, Jewish and South American heritages. Over 180 languages are spoken by residents [32].

### The intervention

Community Protect (CP) is a model of partnership working between the local authority and voluntary and community organisations, who work with the most disadvantaged populations within the area. It was set up during the height of the COVID-19 pandemic, with the original aim to convey up to date information about the virus to these communities, and to improve health protective behaviours including vaccine uptake. This approach, of working with and through small voluntary sector organisations, was built on the premise that these organisations understood, had often grown from and had good reach into different local communities, and were generally known and trusted by the target population. In the aftermath of the pandemic, the borough’s public health team successfully applied for short term (12 months) external funding to plan, implement and monitor a programme of activities to help reduce mental health inequalities. The CP model was adapted as part of this, with a new focus on promoting the mental health and wellbeing of residents.

**Fig. 1:**
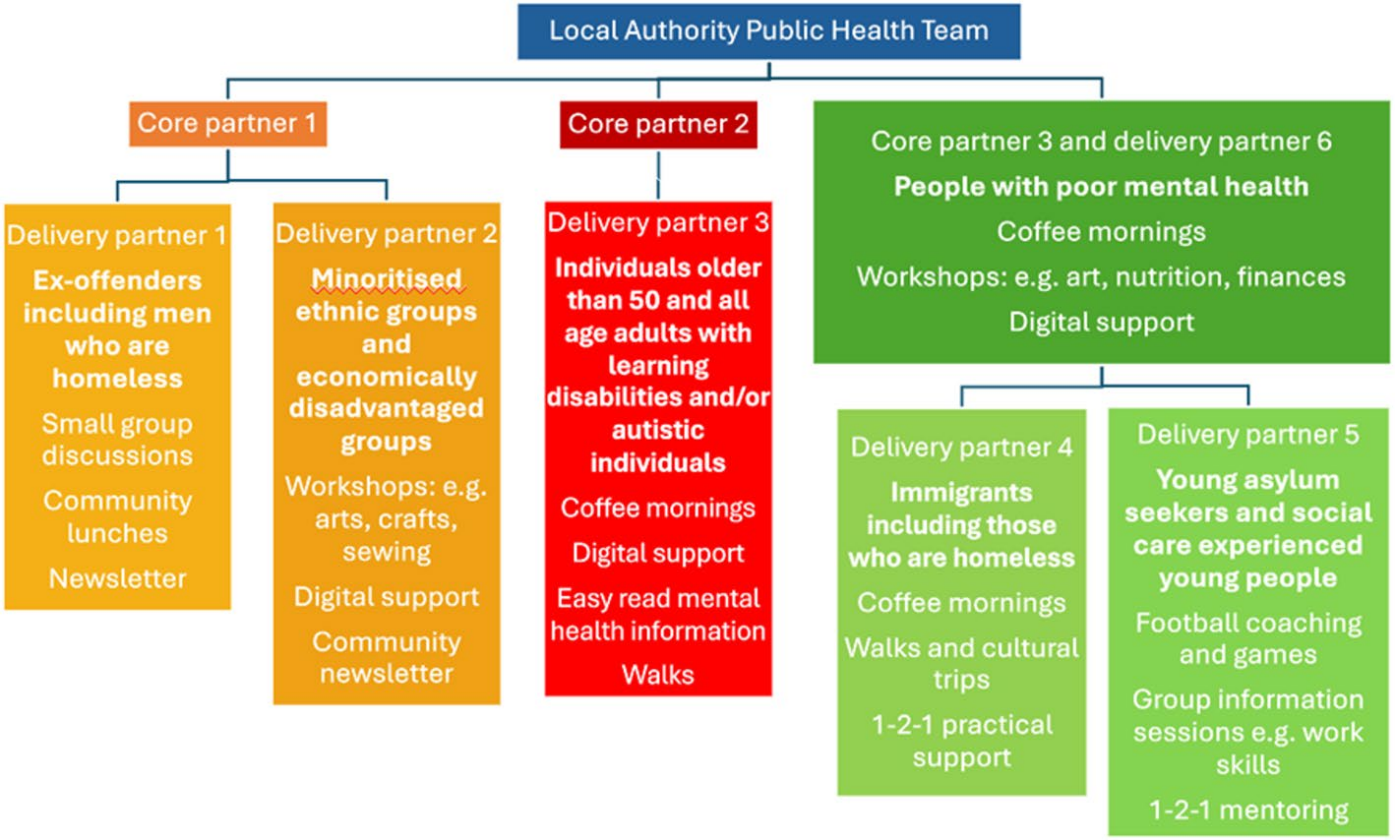
This figure shows the structure of the CP model, included populations and activities provided. Note that core partner 3 was also delivery partner 6

The local authority commissioned CP to work with seven priority groups: Black, Asian and other minority ethnic groups, low-income households, older people, people with existing mental health conditions, people with learning disabilities and autism, homeless people and young people not in education, employment or training (NEET). These groups make up significant numbers in this local authority and were identified as being at risk of poor mental health and also being less likely to access mental health services. A contract was awarded to three larger voluntary sector organisations (the ‘core partners’) who had worked as part of the original CP programme. Core partner 1 was a local charity focused on reducing health inequalities and strengthening communities, core partner 2 was a local branch of a city wide community interest company that uses community engagement to improve neighbourhoods and public services and core partner 3 was a local branch of a national mental health charity. They identified six small, community organisations (the ‘delivery partners’) who worked with one or more of the target populations, decided upon how the funding would be distributed to the delivery partners and arranged this through their own application process. The CP model, including the priority groups with whom the delivery partners worked, is shown in Fig. 1. The core partners provided a bridge between the local authority public health team and the delivery partners, working with them to decide how they would meet the aims of CP in ways that were appropriate for their particular populations.

**Fig. 2:**
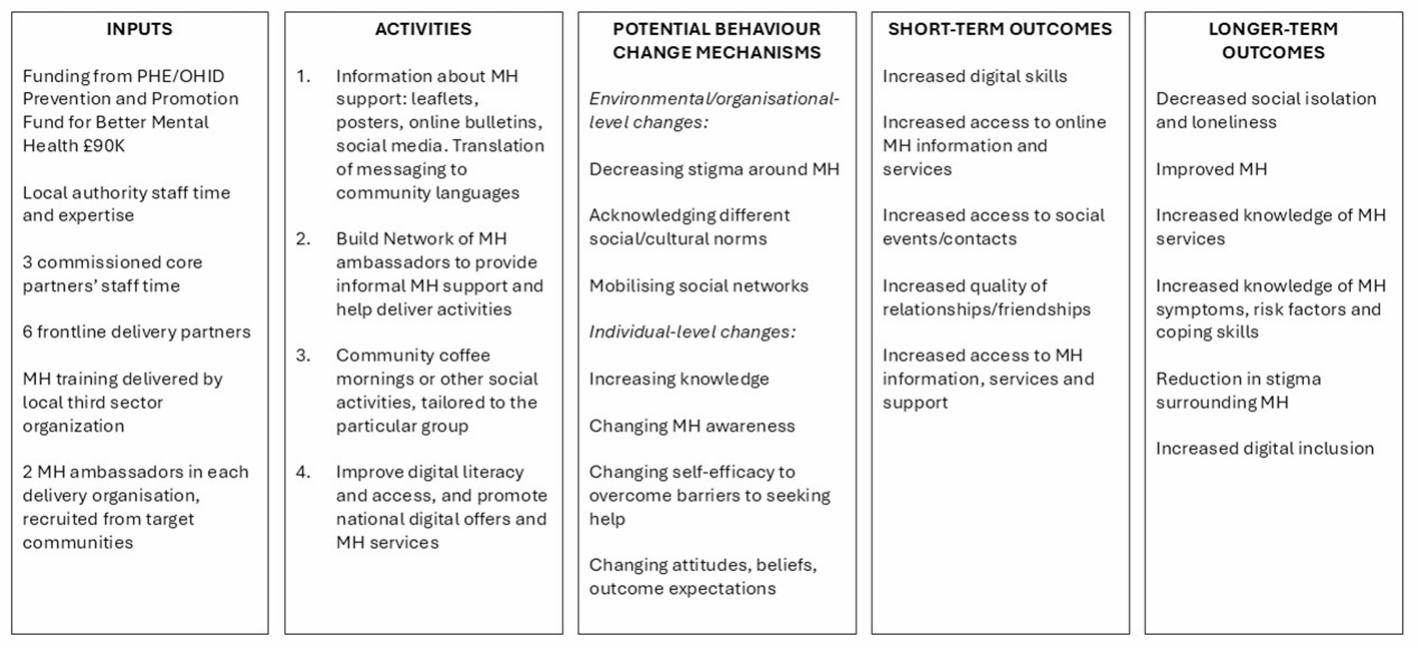
This figure is a logic model that outlines the components of CP, the theorised mechanisms by which it works, and the outcomes aimed for. *MH*  = Mental Health

CP aimed to improve mental health and wellbeing through various mechanisms including reducing social isolation, improving connections between residents, and sharing information about available mental health support and how to access it. The main activities delivered by each delivery partner are shown in Fig. 1; these varied by organisation, and were designed by the organisation leads, based on what their population had found helpful previously, what they said they would like and what worked well when they tried it out. A core component in each organisation was the creation of mental health ambassadors (two per organisation). These individuals were trained in mental health awareness and then supported individuals to take up their organisations’ services. Figure 2 is a logic model that outlines the components of CP, the theorised mechanisms by which it works, and the desired outcomes. CP’s mental health programme ran from September 2021-September 2022, with data collection taking place May-September 2022.

### Data collection

Qualitative methods are well placed to understand the processes and contexts in which community interventions are delivered, and the experience and likely impact of the intervention from the viewpoint of those that are involved [33, 34]. We conducted semi-structured interviews (*n* = 28) with the key local authority staff who had planned the CP partnership model and applied for funding, lead staff working for the core partners on this project, all the lead staff working for the delivery partners and the mental health ambassadors. The number interviewed per organisation varied depending on the number of people involved; delivery partner 3 was an amalgamation of two organisations and therefore had slightly more leads and ambassadors.

Core partner staff introduced the research team to the leads of the delivery partner organisations, and the delivery partner staff introduced the team to the mental health ambassadors. Everybody approached agreed to take part in an interview. One delivery partner did not want to be heavily involved in the research and so only agreed for the organisation lead to be interviewed; no further interviews or observations took place. Another delivery partner had not managed to organise any group activities during the course of the research time period, and so no observations took place. Those who were volunteers were given a £25 high street shopping voucher as a thank you for their time. Interviewees were given the choice of an online, telephone or in person interview: 21 were conducted via Microsoft Teams or Zoom, four by telephone and three in person. We audio recorded all interviews using an encrypted device. We provided participants with an information sheet, and they gave written consent before taking part.

We followed topic guides during the interviews, which covered: the aims of CP, the quality of partnership working, uptake of activities, perceived outcomes for individuals and organisations involved, barriers and facilitators to implementation of CP and—for delivery partners and mental health ambassadors only—recruitment, training, and experiences of the mental health ambassadors. We used the guides flexibly, allowing interviewees some control over the focus on the interview, according to what they felt was most important to discuss.

We also conducted observations of activities in the community organisations. We completed observation schedules, which covered: activity description, health information or support provided, broad summary of attendees, interactions that occurred, role of the mental health ambassadors and barriers and facilitators to the activity running smoothly. This enabled the interviewer to understand the context of each delivery partner organisation, and to triangulate with the interview data. Table 1 shows the final number of interviews and observations by organisation.

The study received ethical approval from the University of Bristol’s Faculty of Health Sciences research ethics committee (ref. 10517).

**Table 1:** The number of interviews and observations conducted

Interviewees	
Local authority (Public Health team, councillors)	4
Core partner leads	
Core partner 1	2
Core partner 2	1
Core partner 3/delivery partner 6	2
Total	5
Staff/volunteers from the 6 delivery partners	
Delivery partner 1	1
Delivery partner 2	1
Delivery partner 3	3
Delivery partner 4	2
Delivery partner 5	1
Total	8
Mental health ambassadors from the 6 delivery partners	
Delivery partner 1	2
Delivery partner 2	0
Delivery partner 3	3
Delivery partner 4	2
Delivery partner 5	2
Delivery partner 6	2
Total	11

Observations	
Delivery partner 1	0
Delivery partner 2	0
Delivery partner 3	3
*Finances information session*,* digital support session*,* picnic*	
Delivery partner 4	1
*Social lunch where one to one advice was available*	
Delivery partner 5	1
*Football training session*	
Delivery partner 6	3
*Two informal coffee mornings*,* art workshop*	
Total	8

We did not formally collect sociodemographic characteristics of the interviewees as selection was based on role within CP. Interviewees were a mix of genders, ethnicities, nationalities and ages. The ambassadors and in some cases the delivery partner leads were members of the population groups that were targeted by the activities.

### Data analysis

We arranged for interview data to be transcribed, and we checked transcripts for accuracy and anonymised them for reporting purposes. We transferred all data to a secure drive only accessible by the study team and we deleted them from recording devices.

For analysis, we undertook a constant comparative, thematic approach, identifying key themes using the Framework Method [35]. First, we coded the interview transcripts by assigning a label summarising the key meaning to each section of text. We grouped codes from the first few transcripts into categories, and together these formed a coding framework. We developed the coding framework iteratively through regular discussion between the first and second authors, as we analysed more transcripts and identified new codes and categories. We based the categories on the interview topic guides where appropriate, but also included new, unanticipated categories where these were more representative of the codes within. Once we had coded all transcripts, we ‘charted’ the data, that is we summarised them by category in a matrix in Excel, where each column represented a category, and each row one interviewee. Cell content was made up of summaries of what each interviewee had said in relation to each category, with exemplar quotations. We did not code the observation notes but charted these on a different matrix, as there were fewer, more overarching categories, due to the less in-depth nature of the data. Once we had charted all the data, the first and second authors brought the categories together into five overarching themes, that addressed the two research questions.

### Positionality and reflexivity

Two authors undertook the main analysis. The first has a background in social policy, welfare, and health, with many years of experience evaluating interventions in the not-for-profit sectors, using qualitative and creative methods. They conducted the coding and charting. The second is a public health academic with expertise in qualitative methods and public mental health. Both discussed the findings throughout the charting process and identified themes that captured the mechanisms and processes within this CP project and the impacts of CP on those involved. Findings were shared with local authority colleagues and core partner organisations to check the validity of the final themes. We used the SRQR checklist when writing our report [Supplementary file 1] [36].

## Findings

Three themes were identified in relation to the process and implementation of the CP partnership model. These captured both the barriers and facilitators to delivery: trust and collaboration, time and resources, and expectations and flexibility. A fourth theme concerned the ambassador role and examined the barriers and facilitators to that part of the model being implemented successfully. A fifth theme, perceived impacts, covered the extent to which the model was perceived to improve mental health, and any other effects. Illustrative quotes have been included – respondents have been numbered, but these numbers do not align with the numbers of the organisations, to preserve anonymity.

### Trust and collaboration

A key feature of the CP model was the reliance on trusted relationships between different actors (Public Health and the three core partners; core partners and delivery partners; and frontline delivery partners and the public):

*“Trust is the biggest single matter or issue really*,* you need to be able to get down to know the people*,* almost at a personal level*,* being able to understand their concerns*,* their fears and being able to work with them through that” [Core Partner 5]*.

The small, community-based organisations who were the delivery partners, were seen to be more trusted by and accessible to marginalised groups than statutory mental health services, ensuring greater engagement and participation in activities, and greater trust in any information provided because it was delivered by them; therefore, trust in these organisations was an important facilitator to the successful implementation of CP.

This trust in some part at least derived from the staff and volunteers in these smaller agencies being drawn from and demonstrating understanding about the target communities, and having an already established record of advocating for their needs and priorities:

*“We are part of them*,* we are rooted and placed in the communities. At all levels*,* all our workers are reflective of the community” [Delivery Partner 6]*.

A number of interlinked factors were found to underpin these trusted relationships and provided a contrast to disadvantaged communities’ experiences of statutory organisations. One factor was the way the delivery partners understood community norms and barriers relating to discussing mental health, including the stigma, shame and fear with which it was often associated:

*“COVID has shown that there are a lot in the communities that struggle through mental health. However*,* we don’t really come forward to talk about it. … not every young black boy or girl or refugee*,* culturally and religiously*,* likes to talk about these things. It doesn’t exist where we come from…You could see there are certain professionals that don’t really understand the needs of those young individuals” [Delivery Partner 3]*.

Much of what these organisations offered was not explicitly related to mental health support but designed to support health and wellbeing more generally through activities such as walks, creative classes, practical support relating to e.g. housing and benefits, sport and communal lunches. This was said to make the activities feel ‘safe’, acceptable and appealing to the target population, providing a further facilitator to successful implementation. Delivery partners and ambassadors reported that it took time for many people to start to open up about their mental health or emotional issues. They often only did so after some time and after they had received help to resolve a more practical issue, such as claiming welfare benefits. This highlighted the importance of helping to address people’s practical needs in creating a platform of trust to enable mental health to be supported.

Another facilitating factor was that those working in the delivery organisations generally spoke the languages of the groups with whom they worked. This created a far more comfortable situation for people to talk about sensitive topics in a less formal way:

*“They speak the same language as them there*,* so there are people to interact with them*,* I think it’s an opportunity for them to talk. Because a lot of them shy away from English and English speaking activities because they’re not quite confident in the language*,* or they feel quite scared or frightened” [Ambassador 5]*.

The final facilitator relating to trust in the delivery partners was that discussing mental health difficulties with organisations generally staffed by members of the same population did not generate the fear of consequences- such as being labelled, inappropriately diagnosed, or involuntarily detained- that reporting problems to professionals in statutory organisations could do:

*“If you’re talking about mental health*,* we all know about the over-medication*,* enforcement*,* too*,* for black people*,* and especially black boys. That hasn’t changed*,* that hasn’t changed at all” [Delivery Partner 6]*.

Trust was also important in the Public Health team’s collaboration with the core partners, as well as in the collaboration between core and delivery partners. Earlier work supporting COVID-19 mitigation and vaccination measures had demonstrated to the Public Health team that the core partners had good knowledge of and connection to the smaller community-based organisations, and could be trusted to support these organisations in delivering health improvement programmes:

*“Trust in the VCSE [Voluntary*,* Community and Social Enterprises] partners being able to deliver*,* which comes over time” [local authority staff 4]*.

This bridging role played by the core partners meant they could shoulder the programme’s administrative, monitoring and reporting requirements on behalf of the smaller delivery organisations, providing a further facilitator to successful implementation. They reported on activities to the public health team, who then reported quarterly to the external funders using standardised monitoring templates. This arrangement protected the smaller delivery partners from having too much resource taken up with these additional tasks, and made the whole process of applying to take part in the first place more straight forward:

*“What I really liked about the application [to take part in CP] was it was not a long application. It was straightforward questions. I know that’s what really puts a lot of grassroots organisations off in terms of funding. It’s the whole writing the bids or the funding application*,* even if you can deliver the work*,* … that puts us off” [Delivery Partner 1]*.

However, there was a downside to using the core partners as intermediaries in this way. Although the public health team and three core partner organisations met regularly to discuss CP, a potential barrier to implementation was that the delivery organisations in CP had little or no contact with the public health team, or with each other. While this minimised the administrative burden, it also meant they had little awareness about the programme as a whole, and were unable to collaborate and benefit from sharing ideas or learning:

*“It was kind of like…you’re out there on your own*,* off you go*,* …that’s fine*,* but if you’re doing something that’s really working and that model*,* can’t we bring that model over here?” [Delivery Partner 4]*.

The lack of a direct relationship between delivery partners that were more representative of the public and the Public Health team, was a contributing factor to a reported lack of consultation and co-production when CP was being developed, which was seen by some as a barrier to CP working as well as it might have done:

*“The better way would have been to say ‘this is what we want to achieve*,* how do you think we can best achieve it?’ It might have saved time because we had to go back and say ‘this doesn’t work’” [Core Partner 2]*.

A key reason for this lack of sufficient collaboration with the delivery partners and the people using their services was the limited time in which CP had to be set up.

### Time and resources

All the interviewees identified time to be a barrier to the optimal delivery of CP. This related to both time to develop and submit the bid for funding, and time for the delivery partners to plan, set up, run, revise and wind down their projects.

The local authority team had only two weeks to design and submit a proposal to secure funding for the 12-month programme of work. The team discussed the programme with the CEOs of the core partner organisations and used learning from surveys and workshops which had been undertaken during the pandemic to inform programme design. However, due to this short timescale, at the time of the proposal submission, only the core partners had been identified and involved and no specific delivery partners. Once secured, the funding was contingent on a very tight delivery timeline of one year. Because of this the core partners had little time to identify and involve the delivery partners, or consult more widely with the local voluntary sector or the public in the programme design:

“*There was never ever a meeting that was about ‘okay*,* how are we actually going to get these people and what is the real aim of this?’… they were just given this money and told do coffee mornings. And that was it…” [Delivery Partner 7]*.

*“If we had more time to work things out and actually consult*,* then perhaps we might have adjusted some of our initiating and start goals*,* it was a mad scramble to engage delivery VCSE … of course there are gold standards ways of doing things that the timeline just didn’t allow for” [Local authority staff 3]*.

As the above quote highlights, this lack of time for planning and discussion with those ‘on the ground’ resulted in several barriers to implementation. Some of the delivery partners already had a plan ready to roll out. But for others it took 2–3 months and in two cases a year to consult with their service users, pilot and test ideas and develop a suitable programme:

*“We did start off with*,* we thought we’d have one big event here. And it didn’t work as well as we would have liked it to have. And so we then reviewed it and then we went back and had a chat to the service users” [Delivery Partner 1]*.

The programme and its timeline was based on the premise that small community organisations are able to be more ‘agile’ in setting up new programmes than larger, statutory services. This was confirmed by several of the interviewees. Some of the organisations were able to begin delivery relatively quickly, particularly where they could extend work they were already doing, or they had a project planned, and this clearly acted as a facilitator to implementation. However, others were critical of the lack of acknowledgement of the costs of developing, planning and running new projects, noting that small organisations do not have spare resources to absorb these, which limits their ability to start up new projects quickly:

*“A lot of these groups needed to get money paid before they could start*,* because in a very small organisation*,* cash flow is a problem…and then some people will require less mobilisation than others because they are already running some activities*,* so all of these factors play into how quick people can start*,* and this is quite often the issue*,* if they then needed to bring extra staff in for that*,* then you’ve got an inevitable delay” [Core Partner 5]*.

Another concern raised in relation to the timeframes was that there was a limit to what could be achieved, even for those organisations that were able to start delivering activities and support quickly:

*“With matters relating to health and wellbeing*,* it takes longer than 12 months to make meaningful changes” [Core Partner 3]*.

Interviewees commented on the time it takes to publicise a new initiative among disadvantaged communities and for them to understand what is on offer and start to accept and use it. Further, there was concern that short-term projects were less effective in addressing long-term, entrenched issues and could in fact be detrimental – by raising people’s expectations and dependence on a service and then stopping it suddenly, while their needs persist:

*“Say you set up a telephone friendship*,* and it can only be funded for so long and people become quite used to that weekly interaction. And then suddenly it’s cut off like that and it stops. And they’re left feeling isolated and abandoned” [Delivery Partner 4]*.

There was a concern that this could undermine the important trust between service users and delivery organisations, if expectations were not met, thereby creating a barrier to the successful implementation of future interventions.

### Expectations and flexibility

Flexibility was discussed both as a facilitator to the implementation of CP, in that it was seen as an advantage that the community organisations had over larger, statutory organisations, but also as a potential barrier because it could create challenges. A key expectation was that CP would support people from the identified target groups who had low to medium mental health needs, but were unlikely to access NHS mental health services:

“*They all focus on a different demographic*,* but the overall objective is much the same. It is to really improve levels of connectivity*,* reduce isolation and loneliness*,* which has obviously been exacerbated during the pandemic for many people … with Community Protect we’re focusing generally on people who’ve got common mental health disorders*,* loneliness*,* or isolation related issues*,* who would not necessarily always present at the GP for IAPT therapy or antidepressants*“*[Local authority staff 1]*.

There was an underlying assumption that individuals with more ‘severe’ mental health needs would be signposted to and able to access statutory and/or more specialist mental health services. In reality, the diverse and generally low-income communities in question experienced many interrelated and layered barriers when trying to access mental health services. Reported obstacles included high thresholds to qualify for NHS support, long waiting lists for talking therapies and other mental health care, and online mental health support being inaccessible to those who were digitally excluded because of low technical skills, poverty and language. In addition, as noted above, many of the communities that were the focus of the CP model held a deep distrust of statutory mental health services, commonly based on negative previous experiences, including racial stereotyping, over-diagnosis, discrimination, low quality care and lack of culturally appropriate care:

*“There were so many barriers to our statutory services*,* our mental health services. There is so much stigma*,* so much discrimination. We know lots of people don’t go. We know that the thresholds are very high*,* waiting lists are high. We need to be doing a lot more in the community. And we think that social networks are an important part of that” [Local authority staff 2]*.

In other words, the barriers to accessing NHS services created a barrier to CP working as intended, as it was difficult for CP organisations to refer on where needed and help people access necessary support. Instead, the community organisations worked as flexibly as they could with anyone who came through the door, whatever their level of mental health need. In some cases this meant these small, non specialist organisations were actually supporting people with serious mental health conditions, which may have detracted from the ‘lighter touch’ work that was intended within the CP model:

*“I’ve got two service users now with PTSD*,* so we need to start looking at working with factors around the challenges associated with their mental health and wellbeing” [Delivery Partner 1]*.

This was not a new challenge for the delivery partners, many of whom were already providing informal mental health support, but participation in CP formalised this expectation.

Other expectations discussed during interviews that potentially created a barrier to successful implementation related to the activities originally specified in CP but that delivery partners had mixed experiences of providing. For example, coffee mornings were intended to be a low-key way to help people socialise, increase participation in group activities, address isolation, and provide some information relevant to mental health, including about local services. One delivery organisation reported them to be a helpful activity that developed the trust required to then undertake advice, counselling and advocacy work, as well as providing a forum to invite guest speakers, who covered topics such as stress and women’s health. However, others felt it had no resonance with and could in fact be seen as culturally inappropriate by their service users. In contrast, they found that sessions tailored to their populations, with a clear focus e.g. aromatherapy, nutrition workshops, pampering sessions or creative arts sessions, attracted larger numbers:

*“I think people know what they’re going to get when they go to an aromatherapy or a nutrition workshop because it is labelled as that. But I think a ‘coffee morning’*,* no matter how we try to tailor it like a ‘coffee for wellbeing’ or*,* ‘come and have a chat in the community’….What is a coffee morning?” [Delivery Partner 8]*.

What became clear through the discussions about the coffee mornings was that CP worked best when the delivery organisations, who had in-depth knowledge of their target population, had the freedom to work flexibly, tailoring what they did to meet that population’s needs. Researcher observations noted good levels of engagement across the CP activities which had generally been tried and tested and built on earlier and ongoing feedback. The planning and delivery of activities was responsive and iterative, often based on issues raised or innovative suggestions made by service users:

*“Now we’re doing evenings*,* because we’ve identified that there are a large number of people with insomnia … people weren’t talking about that before. But now*,* a lot of people are having problems sleeping*,* maybe due to loneliness. I don’t think people actually recognise that loneliness is what a lot of people are stuck with” [Delivery Partner 8]*.

Although the freedom to be flexible and responsive was a facilitator to successful implementation of CP, as noted above, some comments from Delivery Partners and ambassadors indicated that it took much longer than expected to explore what might be useful and acceptable to their service users. This again highlighted the importance of allowing enough time to work collaboratively when developing interventions, otherwise the time needed to work flexibly became a barrier.

###  The ambassador role

There were mixed experiences relating to the mental health ambassador role and the barriers and facilitators to this part of the model being implemented successfully. The background and experience of the ambassadors, and how they came to be involved, varied. In most cases those who became ambassadors were from the target user group, which had been an original intention and was seen as an important facilitator:

*“For the ambassadors it’s been great to continue to raise the awareness around mental health and being able to communicate it in their own words to their peers” [Delivery Partner 3]*.

But in two organisations the role was advertised externally, and the ambassadors recruited were not ‘peers’ of the service users.

The work the ambassadors did varied between organisations, ranging from practical and administrative duties to more skilled requirements such as facilitating discussions and giving one-to-one mental health support:

*“We have adapted it for the people we support. I wouldn’t be expecting [ambassadors’ names] to be able to recognise poor mental health and some elements of that*,* I wouldn’t expect them to do the direct signposting*,* I would expect them to come to me*,* or someone in their support circle to say ‘I’ve got this person*,* I think they need some help’” [Delivery Partner 8]*.

Therefore flexibility in how the role was implemented was important both to meet the needs of service users, and also to protect the ambassadors from unreasonable expectations. The majority of ambassadors reported enjoying and benefitting from the role, indicating that this flexibility was enacted successfully (see Perceived impacts below). However, a minority indicated that they sometimes found the role unclear, frustrating and challenging, not least because of their awareness of its potential importance:

*“It’s quite an important make-or-break role when you have people that are struggling with mental health issues*,* who have somehow summoned the courage to come and meet a complete stranger or group of strangers with the indirect admission that they’re struggling mentally. I call it a make-or-break role*,* because at that point you may never get them back there*,* if they don’t receive a decent reception and there is the empathy and compassion that they need” [Ambassador 2]*.

In one case, an ambassador stepped down because they found it more challenging than anticipated and in another, the delivery partner lead felt an ambassador had turned out to be unsuitable. This left them with the challenge of trying to manage this sensitively, and balance different needs:

*“it’s not just about personality*,* it’s about the approach and skill. Because it’s not for everybody… I’ve realised now*,* people know when people are being genuine. They tell you that. ‘Oh*,* I don’t really want to talk to her’” [Delivery Partner 4]*.

While the agreement between the local authority team and the core partners was for all ambassadors to undertake an approved introductory mental health training course, not all had done so by the time of interview.

These challenges of ensuring ambassadors had the necessary skillset to begin with, and received any further training and support needed, therefore created a potential barrier to the role being implemented successfully. It is interesting that none of the interviewees raised the voluntary nature of the role as a barrier to its successful implementation, but it is possible that there would be less interest in an unpaid role, making it potentially more challenging to recruit suitable people.

These discussions about the challenges connected to the ambassador role highlighted the need to discuss and agree clear parameters with delivery partners, and to ensure adequate and consistent training and support was available, with clear guidance about who would be suitable for the role. When this was done well, positive impacts for both the ambassadors and the service users were seen, as discussed next.

### Perceived impacts

Our interview findings indicated participants at all levels of CP had benefitted. Local authority interviewees reported that working with a range of community organisations, who were well embedded into some of the most disadvantaged populations in the area, vastly improved their reach to those populations. This provided an important avenue for individuals’ mental health needs to be recognised and met in ways that were culturally tailored and acceptable to them, and for them to be supported to access other services as appropriate:

*“Because we are not going to know what a Somali man might want to attend*,* what kind of activity they might want to attend. Because they know. The Public Health team are not close enough to people not in education*,* employment and training. We are not going to know what they want to attend” [Local authority staff 2]*.

*“We really wanted this to build on elements such as trust*,* community connection and have trusted messages from trusted sources*,* coming from grassroot levels. So we wanted conversations about mental health to happen really naturally*,* whether that was in a mosque*,* or in a car park*,* or in a home*,* but fuelled with facts” [Local authority staff 3]*.

The Public Health team appreciated the value of developing ongoing trusted relationships with third sector organisations as a more sustainable and effective way to improve the health of marginalised groups and reduce inequalities.

Similarly, the three core partners felt this programme had helped connect them to and strengthen partnerships with grassroots groups, which they saw as an important long-term benefit:

*“It leaves a legacy of working with community groups who have built more trust not just with us but I would suggest with Public Health and the council as well. And that is quite a strong thing*,* particularly given the pandemic and the high level of mistrust that came through” [Core Partner 5]*.

The delivery partners reported a number of benefits that they experienced as organisations and as individuals. One lead said they had cried when they were told they had been granted this funding, as it meant they could address some of the mental health needs they had already identified in their community and try out initiatives they had wanted to do, but lacked the funding for previously:

*“Thanks to the project we could start doing things that we had in our minds for such a long time but because of the funding we were unable to start” [Delivery Partner 5]*.

These lead individuals were hugely invested in the organisations and projects they had created and felt a great sense of responsibility to their communities and service users. Some reported using their own personal funds prior to CP to keep things going, in addition to giving their time for free. Unreliable funding or worrying about project continuation caused immense stress, as well as limiting the work and its potential impact.

Most of the delivery partners were hoping and planning to continue supporting the mental health of their service users as a result of the awareness, skills and knowledge they had gained through this programme. Many hoped that being part of CP and demonstrating their work and worth would help them access more funding and opportunities in the future:

*“I did a presentation to the Mayor of London on the work that we do on mental health and he straightaway said ‘I want you to be part of the recovery plan for young people in London’”[Delivery Partner 3]*.

*“How can we implement this across the entire organisation? You know this isn’t a 12-month project for us*,* this is something that is going to be incorporated within the organisation’s culture” [Delivery Partner 8]*.

Despite challenges associated with the ambassador role discussed above, several benefits were highlighted. The ambassadors discussed becoming more confident, seeing improvements to their own mental health and wellbeing, developing transferable skills such as group facilitation and public speaking, gaining ideas in relation to future career choices and developing greater understanding of mental health and the needs of their community:

*“Just listen. Just open your heart and just care about these people. You care about them. Really pay attention to them in that way…purely from a self-interest it was a good practice putting compassion into action” [Ambassador 2]*.

*“It definitely improves my mental health because I am not stressing about A Levels and I have something to do. I have a purpose. I’m not just wasting my summer” [Ambassador 4]*.

These benefits are important to note, given that ambassadors were not paid for this role and it is important to ensure they are not being relied upon to help deliver the model, without receiving anything in return.

Ambassadors and delivery partners also observed benefits among service users including improved connection and companionship, the chance to engage in enjoyable activities and learn new skills, timely and accessible support for their mental health, and practical support in completing forms and accessing other agencies:

*“There’s this sense of closeness from everybody sharing*,* and people just feel a sense of connection and happiness when that does happen” [Ambassador 3]*.

*“I’ve had people who have phoned up and said to me ‘I can’t cope anymore*,* I’m at the end of my tether’. And by the end of the conversation*,* they’re laughing and joking and they’re saying I’m so pleased I spoke to you” [Delivery Partner 5]*.

There was a sense that what these grassroots organisations offered could often seem small, but could nonetheless incrementally make a big difference to someone’s life:

*“What might not look significant to us is actually quite a lot*,* just coming out*,* mixing with people is quite a lot” [Core Partner 2]*.

These low key, cumulative impacts, along with the flexible nature of CP already discussed, made evaluation based on measurable outcomes a challenge. National funders of CP had stringent monitoring requirements to measure the impact on mental health using validated questionnaires and requested numbers and demographic details of participants. Although interviewees understood the importance of monitoring CP’s reach and impact, the methods used were seen by many to be disproportionate, too time consuming and inappropriate for evaluating the diverse, ‘light touch’ and flexible activities being delivered. Further, it was noted that delivery partners rarely had the staff capacity to dedicate the time required for monitoring and evaluation. In many cases one or two paid staff fulfilled all functions:

*“[Funders] need to walk 100 metres in somebody else’s shoes … They need to properly understand*,* when we say ‘frontline delivery groups don’t have the capacity to collect data in the way that you want it’. That’s a line I trot out quite regularly*,* but they need to understand what sits behind that in a very real sense” [Core Partner 1]*.

## Discussion

### Summary of findings

Our qualitative evaluation of CP, a community-based mental health partnership model that targeted groups who are disadvantaged, identified a number of factors that were important for its successful implementation; see box 1 for a summary of these and their practical implications.

**Table:** Box 1: summary of the facilitators to the CP model being implemented, and implications for policy and practice

Facilitators to the delivery of CP and implications for policy and practice
•* Delivery partners provided an approach to mental health support that was trusted because it was culturally acceptable, accessible, safe and delivered in the language of those receiving the support* Mental health services should consult with and learn from small community-led organisations about how to ensure they are acceptable, accessible and welcoming to different minoritised and disadvantaged communities
•* The local authority public health team trusted the core partners to identify and support the most appropriate community-led organisations to become delivery partners, and the core partners trusted the delivery partners to effectively delivery to the communities and groups* Statutory bodies should invest time and resource building up trusted relationships with larger VCSE organisations or networks, as they can provide an effective ‘bridge’ to connect with underserved communities
•* More time to apply for the funding, and more time to set up the CP model would have allowed for better consultation with the delivery partners and their populations, and more time for the delivery partners to plan their work* Funders should provide realistic timescales to apply for funding and deliver projects, to enable engagement with community-led organisations and ideally co-production of plans
•* Adequate time and resourcing are needed to enable small grassroots organisations to publicise new interventions, encourage participation and see health-related changes* Funding for small community-led organisations should be longer term to allow them to design and co-produce new interventions to meet emerging needs and have an impact
•* Delivery partners’ flexible ways of working meant that they could respond to a wide range of mental health needs, which sometimes included serious mental health conditions* The barriers to disadvantaged, at-risk groups accessing mental health services for serious mental health conditions need to be removed, so these can be accessed alongside emotional and practical support from non-specialist community-led organisations
•* The ability of delivery partners to review activities and tailor them to their populations meant that the CP model was able to meet the needs of a range of target populations* Small, community-led organisations should be acknowledged and resourced as an integral part of the mental health support system. Models must acknowledge them as the experts in what works for their populations and be designed to allow flexibility in what is delivered.
•* Mental health ambassador roles can be a way of connecting with and generating trust among underserved populations, providing important peer support and benefitting the ambassadors themselves* Where ambassador or peer support roles are used, these must be provided with adequate training, supervision and support

Firstly, relationships had already been built during the COVID-19 pandemic between the public health department and the core VCSE partners. This supported the rapid establishment of this new programme focusing on mental health. Further, the fact that the intervention made use of existing trusted relationships between the core partners and the smaller community organisations facilitated the model getting off the ground and being delivered in the limited timeframe given (12 months) and maximised its reach into the target populations. It takes time and communication to build up the necessary trust for such collaboration, but once this has been done, the evidence here indicates that such partnerships have the potential to work beyond one off interventions but can go on to address new and emerging health issues.

Secondly, time was a key factor. The public health team was required to submit an application within a two-week window for the external funding. This was a very short time frame, particularly in the context of their other workstreams and priorities. Although the application was successful, the short application window limited detailed planning and scope to collaborate with delivery organisations and the public, to help ensure that the model was appropriate and relevant to their needs. Equally, the twelve months allocated to set up and deliver diverse, innovative programmes was too short to achieve as large an impact as possible. This barrier was exacerbated by the fact that the delivery partners as small grass-roots organisations did not always have the resources to plan ahead. Our findings highlight the practical difficulties experienced by local authorities when bidding for external funding and commissioning, operationalising and monitoring community-based programmes, over a short timescale. The setting of unrealistic expectations by statutory bodies does little to enable the establishment of trust with voluntary sector organisations. Lengthening the time allowed to apply for, plan and implement new programmes would enable greater collaboration, more effective interventions and removal of some of the barriers identified in this paper.

Thirdly, it was important that the community organisations were given flexibility to tailor the CP activities to meet the needs of their particular service users, and to adapt what they did over time in response to experience, issues emerging and feedback. This helped ensure that what they delivered provided the best support for mental health and wellbeing among the disadvantaged groups with whom they worked. However, the tension between the short time frame and the need for flexibility did sometimes mean this became a barrier to plans being implemented before the end of the 12 months.

Our findings show that a partnership model, in which Public Health teams work in partnership with large VSCE organisations to support small frontline, community organisations, has the potential to make an important contribution to improving mental health and reducing health inequalities, particularly for those groups whose needs do not meet the thresholds for statutory services or who cannot access services due to lack of availability or acceptability. However, there are risks involved; if funding is short term and the community-based support is time limited, this may erode trust between the public and the delivery organisations and undermine potential benefits. And if interventions are not developed in partnership with delivery organisations and the public, then activities provided may not be relevant or acceptable. Successful implementation of models such as CP relies on strategic planning, informed and rooted in effective collaborative working, commitment between partners at different system levels and across organisations, realistic timescales and adequate funding for an appropriate duration.

### Limitations

Our study was limited to one geographical area in the UK; therefore it is unclear how generalisable our findings are. However, the barriers faced in terms of accessing early mental health support are likely to be similar in many other urban areas that have higher levels of economic and social deprivation where there are diverse and underserved population groups. We did not include service users as participants in the study because our main question was regarding the implementation of this programme model. This means that our reported findings about benefits to service users reflect the views of those providing the services, which includes some individuals from the same communities, but not the views of those receiving the services. Future research should include the views of those targeted by such partnership models regarding the impact on health and health inequalities from their perspectives. A final limitation related to the fact that the CP model and activities that it involved was continuously iterating, as is often the case with new, innovative and responsive services. This meant it was hard to capture the full extent of its implementation and likely impact through interviews that were conducted at one point in time.

### Contribution to the wider literature

Within the limited literature available on this topic, the majority of community-based mental health interventions involve older adults and those from a minority ethnic backgrounds [30]. Our findings confirm the value of support within the community for these populations, but also for other groups at risk of poor mental health, including young asylum seekers and refugees with few social or local connections who are at risk of being out of education and work, ex-offenders at risk of homelessness and people with learning disabilities. Several models of community-based support have been explored in the literature, including social prescribing [21], co-location of services [15], and therapy/support delivered by lay workers [37]. We extend this body of literature, by demonstrating the value of collaboration between statutory Public Health teams and large third sector organisations, to support small, grassroots organisations in their work with marginalised communities. Themes identified in this research, such as flexibility, trust, and the importance of realistic timelines, align with broader findings in the literature on effective partnership working [27, 28]. An evaluation of mental health alliances in England also found that VCSE collaboration is built through trust and that having necessary time is essential to establish clarity about a shared vision and levels of commitment from partners [38].

CP was delivered in the context of diverse and low-income communities experiencing layers of interrelated barriers in accessing suitable mental health support, combined with a deep mistrust of statutory services. This mistrust is commonly based on negative previous experiences. including racial stereotyping, which has been attributed to structural racism and a lack of cultural competency in the healthcare system [39]. For example, Black Caribbean and Black African individuals with psychoses are more likely to have contact with the police and the criminal justice system yet are less likely to have GP involvement compared with White populations, and all Black populations are more likely to be admitted to civil and forensic detentions under the Mental Health Act [40]. The importance of structural inequalities and discrimination as determinants of health including mental health in the UK has been well documented [9, 18]. We found that small, grassroots organisations, when funded and supported by larger organisations, can play a vital role in addressing these challenges by providing trusted, flexible and more appropriate support. Gater et al., (2010) [23] similarly found that providing culturally appropriate opportunities to engage in social activities as part of a primary care based mental health support intervention was an important element to its success. Molenaar et al., (2024) [25] also identified culturally sensitive support as a key strength of community organisations in supporting migrants’ mental health in Belgium. Third sector keyworkers supporting the wellbeing of older adults in England highlighted that having a shared cultural identity and local community connections can be more important than just speaking the same language when engaging with citizens whose first language was not English [41].

Workers in these organisations often reflected the diversity of the communities they served and had an awareness of the stigma surrounding mental health issues in these communities, much like the delivery partners in CP. Consistent with this study’s findings, Molenaar et al. [25] also concluded that community organisations were well-equipped to support individuals with a broad spectrum of wellbeing needs and could tailor their approach to suit each person’s unique circumstances. However, the impact of such interventions is likely to be modest and cumulative, therefore without sufficient long-term funding and improvements to other parts of the wider mental health support system, there will be a limit to how far mental health will be improved at a population level [42]. Within the CP model, the small community-based delivery partner organisations were only ever intended to support people with low to medium mental health needs, signposting to other services where necessary. But such models can only work if there are sufficient available, accessible and acceptable services for those with more severe mental illness [21] and clear, equitable pathways for all communities to access these.

A key aspect of the CP model was the mental health ambassador role, to provide information, support and signpost people to other services. Each delivery partner operationalised this role slightly differently, but in most cases, the ambassadors were volunteers, and were drawn from the groups served, with the idea that this would facilitate informal and open interactions that were comfortable for service users. Other studies have highlighted how peer led mental health support can be acceptable and beneficial [37, 43]. An umbrella review of peer support approaches for mental health found some evidence that peer support can improve depression symptoms, and increase self-efficacy and recovery [44]. Benefits for both the ambassadors and service users were identified through interviews, with benefits to service users including improved connection, opportunities to learn new skills and gain practical support. Ambassadors often reported an increase in confidence and developing a greater understanding of mental health needs in their community. However, our findings highlighted the challenges related to having minimally trained, unpaid workers take on such a role. Ambiguity of the role, unclear boundaries, and organisational issues such as lack of support and training have been identified as important factors influencing the effectiveness of peer support approaches in mental health [44]. The importance of ensuring sufficient training, support and clarity of role for peer supporters has been highlighted elsewhere [45]. Adequate training, mentoring and supervision for mental health ambassadors can lead to a high level of satisfaction with the role, increases in mental health literacy and decreases in overall mental health stigma, as demonstrated in Molewyk Doornbos et al.’s (2025) [46] study involving Michigan residents who were trained to become Lay Mental Health Ambassadors in their communities.

It was clear that a balance needed to be found between ensuring ambassadors were perceived as peers by service users, while ensuring that they had the capacity and skills and were themselves well enough to work with vulnerable and often distressed people and prioritise their needs. This echoes findings from elsewhere about the scope, strength and challenges of using volunteer support for vulnerable families [47].

Our findings revealed the difficulties of measuring effectiveness of local, small-scale interventions such as CP in ways that are acceptable to community groups. The separate evaluation using quantitative measures produced for the funders did show improvements in mental wellbeing, but our interviewees described the burden that this entailed. Light touch methods are likely to be better suited to community-led interventions, where activities develop over time, are generally delivered on a drop-in basis, and impact is likely to be modest and cumulative, but these are lacking in the literature [48]. Future research should examine the best ways in which to evaluate such interventions, that meet funder requirements, provide robust findings, and are also acceptable to different and more marginalised population groups. This should include examination of the cost effectiveness of such interventions, as such information is vital to help persuade funding bodies to increase resources over the longer term.

## Conclusion

Small, third sector organisations, with the support of larger ones, can be an important part of the mental health support system, particularly for disadvantaged groups and those who experience barriers to statutory support. The key role of such organisations needs to be adequately acknowledged by local and national policy-makers in terms of funding, relationship building, support and opportunities to co-produce interventions. But they can only be part of the solution, and they need to operate alongside statutory services that are accessible, non-discriminatory, equitable and culturally acceptable, if population mental health among the most at-risk groups in society is to improve.

## Supplementary Information

Below is the link to the electronic supplementary material.

Supplementary Material 1.

## Data Availability

The data that support the findings of this study are available from the corresponding author upon reasonable request.
